# Novices may be trained to screen for abdominal aortic aneurysms using ultrasound

**DOI:** 10.1186/1476-7120-11-42

**Published:** 2013-11-22

**Authors:** Anh TV Nguyen, Geraldine B Hill, Matthew PT Versteeg, Ian A Thomson, Andre M van Rij

**Affiliations:** 1 Department of Surgical Sciences, Dunedin School of Medicine, University of Otago, PO Box 56, Dunedin, New Zealand

**Keywords:** Abdominal aortic aneurysm, AAA, Screening, Novice, Ultrasound

## Abstract

**Background:**

Highly trained vascular sonographers make up a significant cost of abdominal aortic aneurysm (AAA) ultrasound screening. However, they are over-trained for this very limited task. Others have reported that health workers (e.g. emergency room staff and nurses) with far less training may be able to perform these scans. The national AAA screening programme in the UK uses staff with limited training. Whether individuals without a health professional qualification could be trained to perform the scan accurately to improve cost-effectiveness is not known. We aimed to investigate whether a short, well-supervised course in ultrasonography could train novices to detect AAA for screening purposes.

**Methods:**

Three novices were trained by an experienced sonographer for 15 days to perform abdominal aortic ultrasound examinations and detect AAA using a portable ultrasound system. The examination included four anterior-posterior aortic measurements: a maximal diameter in the coronal plane and three diameters of the suprarenal, mid and distal infrarenal aorta in the transverse plane. The novices independently scanned 215 subjects following training; experienced sonographers repeated the measurements on the same subject in the same session. Using Bland-Altman plots and CUSUM analysis, the novices’ and experienced sonographers’ accuracy and efficiency measurements were compared. Factors influencing performance were recorded.

**Results:**

The novices measured the maximal coronal aortic diameter accurately, to within 0.46-0.52 cm of the true diameter; 85-97% of their coronal measurements were within 0.5 cm of the assessors; kappa statistic and Bland-Altman plots show a high agreement with the assessor’s measurements. However, the novices’ measurements of the three diameters in the transverse plane were outside clinically acceptable limits. Assuming a referral policy for a second scan for scans recorded as 'difficult’, only one novice missed a 3.13 cm aneurysm.

A CUSUM quality improvement analysis demonstrated substantial improvements in the scanning efficiency of the novices with continued scanning experience.

**Conclusion:**

This study showed that novices could be trained to screen for AAA over 15 days. However, the need for continuing quality improvement is critical, especially in more technically demanding cases. Measuring the maximal infrarenal diameter instead of specific segmental diameters may be more appropriate for AAA screening using novices.

## Background

Results from randomized controlled trials have suggested that screening for abdominal aortic aneurysm (AAA) is beneficial and cost-effective [1,2]. Based on this evidence, screening programmes for AAA have been implemented in several countries. Recently, the United Kingdom AAA screening committee reported a detection rate of 1.7-1.8% in the first two years, which was much lower than the anticipated 4.5% to 7.7% from four large randomized trials [3]. A possible reason for this is that AAA incidence may be decreasing worldwide [4], which raises the question of whether AAA screening will remain cost-effective in the future. This information is particularly important to countries that are considering implementing a national screening programme for AAA, such as New Zealand.

Ultrasound detection of AAA is said to be a relatively simple and easy process [5] and so screening may conceivably be performed by less extensively trained staff who require only a short training period, making the practice more cost-effective. On the other hand, effective AAA detection relies on the accuracy of the ultrasound operator at measuring the aortic diameter, particularly for small or borderline aneurysms. Less skilled operators may not measure the aorta to a clinically acceptable level of accuracy. It is therefore important to determine what level of ultrasound skill and experience is required for accurate AAA detection.

The literature on this topic is currently limited. In 1998, Singh et al. [6] demonstrated that a radiologist and two cardiovascular nurses given two months of ultrasound training could subsequently detect AAA with accuracy. Two pilot studies concluded that less skilled people such as medical students and emergency residents could screen for AAA after a short ultrasound course [7,8]. We postulated that people with even less medical and imaging experience might be trained for this task. This study aims to investigate whether three novices, after 15 days of ultrasound training, could reliably detect AAA. To achieve this, the accuracy and efficiency of aortic measurements by the novice trainees was compared with that of more experienced vascular sonographers.

## Methods

### Settings & patient recruitment

This study was a double-blinded prospective longitudinal study conducted over the 4-month period between November 2010 and February 2011. Participants aged 50 years and above were recruited from the public and from patients referred to the Otago Vascular Diagnostics laboratory. The participants were instructed to avoid smoking and “gassy” foods on the day of the scan to minimize bowel gas. Written, informed consent was obtained having Ethical approval from the regional ethics committee (Lower South Regional Ethics Committee).

### Novices

A group of three novices were recruited based on their previous lack of any imaging experience or training in ultrasound. The group consisted of: Novice 1, a second year (preclinical) medical student; Novice 2, a newly employed, inexperienced vascular technologist; and Novice 3, a Physical Education graduate. All had tertiary level health science education. Following the completion of the detailed initial study, a further two subjects were recruited and assessed using similar quality control measures.

### The assessors and trainer

Five experienced vascular ultrasound technologists working in the Otago Vascular Diagnostics Laboratory, in Dunedin, operated as the assessors against whom the novices were compared. The most experienced member of this group, with 16 years of experience in vascular ultrasonography, instructed the novices during the training phase of the study.

### Training

The 15-day training course consisted of a theoretical and a practical component. The theoretical component included didactic teaching sessions on the principles and techniques of ultrasonography, the anatomy of the abdominal aorta, the hemodynamic principles of arterial blood flow as well as AAA pathology and epidemiology. For the practical component, the novices learned to use an ultrasound machine, to acquire the static image of the abdominal aorta and to measure its diameter. A standardized protocol was implemented that involved measuring four anterior-posterior aortic diameters: a maximal diameter in the coronal plane and a suprarenal, mid infrarenal and distal infrarenal diameter in the transverse plane*.* The diameters were measured from the outer wall to the outer wall with electronic calipers on screen with the images captured in systole. We defined an AAA as one with an infrarenal diameter of 3.0 cm or more. The novices practiced measuring the abdominal aortic diameter one-to-one and in a group under supervision, and developed further experience during self-directed and peer-directed practice. The trainer gave performance feedback during approximately 30 supervised sessions as well as feedback based on the stored images collected. On average, each novice had practiced performing 50 ultrasound examinations, which included patients with or without AAA.

### Equipment

The novices used a portable laptop-based ultrasound system, Terason with the following features: 3.5 MHz curved array transducer, 25 cm penetration depth, time-gain-compensation, B-mode, real-time imaging, “cineloop” and depth control settings. These features are equivalent with the standard ultrasound system, Antares used by the experienced sonographers in a clinical setting.

### Screening and quality assessment

Once the 15-day training period was completed, the novices performed screening with no further feedback for the period of the study. The three novices measured each participant’s aorta independently following the standardized protocol and were compared to those obtained independently by an assessor. Each novice and assessor recorded the scanning duration, and the level of difficulty obtaining each image on a scale of 1–4, with 1 indicating the scan was not difficult, and 4 that the scan was very difficult, with a sub-optimal image and the patient should be referred for formal screening at the Vascular Laboratory. The number of scans from which no image could be obtained was recorded.

After each ultrasound examination, participants completed a questionnaire to provide cardiovascular risk information and demographics; fasting status, height, weight, and waist and hip circumferences were also recorded.

### Statistical analysis

The repeated measurements by the novices and the assessors were compared. The variability calculated as 1.96 times the standard deviation of the mean difference, signified how much each novice’s measurements varied from an assessor’s. This variability was compared with a clinically acceptable difference (CAD) of 0.5 cm, described by Jaakola et al. [9]. The 95% limits of agreement (LOA), calculated by the sum of the mean difference and the variability, is the range within which 95% of the differences between the novices’ and the assessors’ measurements lie. These are illustrated using the Bland-Altman plots in which the differences between measurements of each novice-assessor pair are plotted against the averages of those measurements. The proportion, measured in percentages, of the novice’s and the assessors’ measurements that were within 0.5 cm was calculated, as described by Lederle et al. [10]. Kappa-statistics were used to determine the interobserver agreement of aortic dilatation (≥ 3 cm) between each novice-assessor pair.

To evaluate the novices’ scanning efficiency, the time taken to complete the scans was retrospectively analysed using the Foresee cumulative summation (cusum) calculator [11]. Two successful time outcomes were investigated: scan times within 5 minutes and scan times within 10 minutes. The weights for success and failure were calculated so that, if the process is set at a predefined success rate, the cumulative score remains close to zero. Limit lines were calculated as described by Gupta et al. [12] that when crossed, signal when a performance has a higher or lower success rate than expected.

Continuous variables are presented as mean ± SD, unless specified otherwise, and were assessed using t-Tests with Welsh correction. Variances were analysed using the F-test and binary outcomes were tested using Fisher’s exact test.

## Results

During the screening period novices and assessors scanned 215 participants. There were 87 men and 128 women. The median age of men was 64 (range 50–86) years and for women was 62 (range 50–105) years. The average BMI was 28 ± 4.7 kg/m^2^; waist circumference 93.7 ± 13.4 cm; 30% were obese (BMI ≥ 30 kg/m^2^). Regarding fasting status, 74% consumed foods within 4 hours prior to their scans. One novice completed only 182 examinations with absence due to sick leave.

### Novice performance assessment

**Figure 1:**
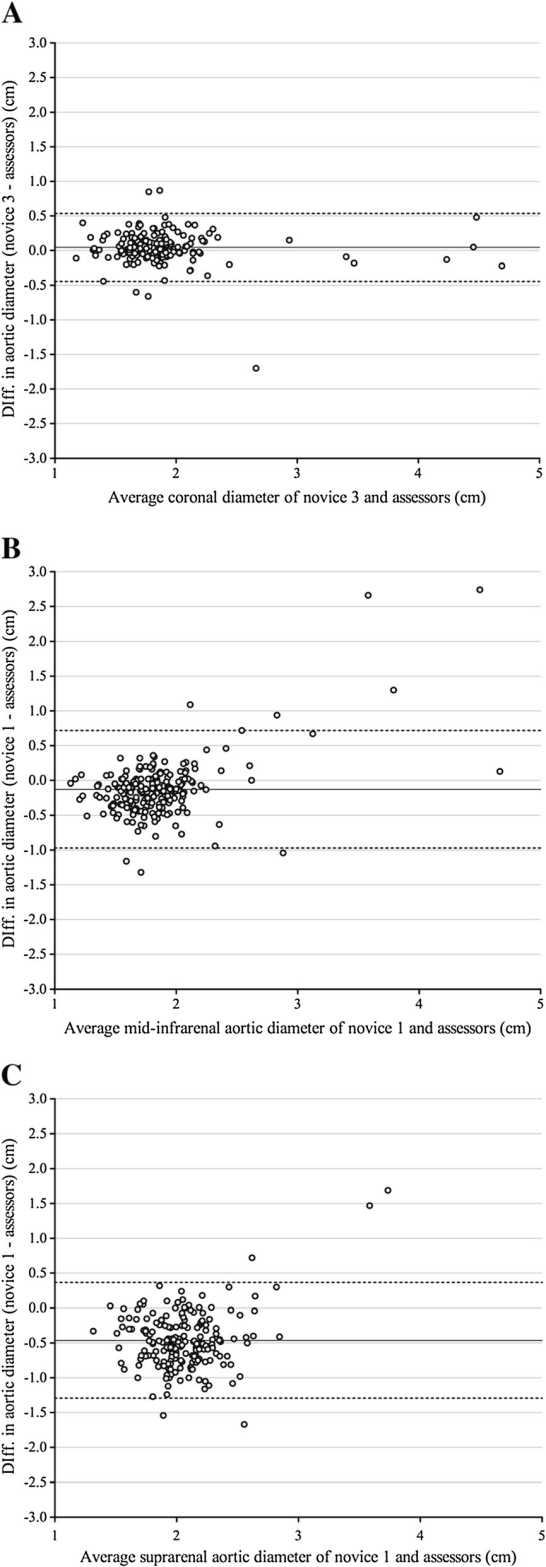
**Bland-Altman plots.** Illustrating variations in performance. Bland-Altman graphs plot the inter-observer differences of the novice-assessors *(y-axis)* against the averages of those measurements. The dotted lines demarcate the limits of agreement (LOA), which should ideally lie within the clinically acceptable difference (CAD) of 0.5 cm. **A** demonstrates an acceptable performance by Novice 3 when measuring the maximal coronal diameter, with no measurement bias and the LOA within the CAD. **B** shows unacceptably high variability in Novice 1 measurements from mid-infrarenal aortic section with outliers in both normal and aneurysmal aorta. **C** shows a large under-sizing bias of 0.5 cm by Novice 1 when measuring the suprarenal segment of aortae, in addition the variability of measurement exceeded the CAD.

a. *Interobserver differences:* The mean differences and the variability and LOA for the measurements for each novice at each aortic section are presented in Table 1. A high percentage of novice observations were within the CAD of ± 0.5 cm. The most accurate measurements by the novices were the distal infrarenal 94% (91-97%) measurements and maximal coronal 92% (88-96%), as illustrated by Figure 1A. In contrast, measurements taken in the transverse plane of the mid infrarenal aortic section exceeded the clinically acceptable range of variability as illustrated by Figure 1B. The poorest performance was in suprarenal measurements of the aorta, where novices had an under-sizing bias of 0.5 cm, variability outside of the acceptable limits, with only 62% (56 - 68%) of observations within the CAD, overall a poor performance as illustrated in Figure 1C.

b. *Strength of measurement agreement:* Novices had very high levels of agreement with assessors in the diagnosis of infrarenal AAA, with all novices having a Kappa coefficient greater than 0.8. Novices had no agreement with the assessors in the identification of localised suprarenal aortic dilatation*.*

c. *Aneurysm Detection:* The assessors identified ten infrarenal aneurysms, which ranged in size from 3.0 to 4.8 cm. Novice 1 missed three of these aneurysms, while Novice 2 and Novice 3 missed one each. All three novices missed a 3.5 cm AAA from the same patient. Novice 1 missed a second AAA that was measured by an assessor at 4.3 cm. In both these cases all the novices graded the scans as a four on the difficulty scale, which would have resulted in these patients being referred back to a more experienced technician and re-evaluated. The third AAA missed by Novice 1 was a small 3.1 cm eccentric aneurysm with a localized area of dilatation. The examination was rated “not difficult”, which was inconsistent with the rating by the assessor and a peer novice (the third novice was not involved in measuring this aneurysm) and thus would not have been referred back for re-evaluation.

**Table 1:** The mean difference and variability of novice measurements of the abdominal aorta

**Aortic measurement**	**Novice 1**	**Novice 2**	**Novice 3**
*Suprarenal*			
Mean difference	-0.46 (-0.52, -0.41)	-0.36 (-0.42, -0.31)	-0.32 (-0.37, -0.27)
Variability	0.81	0.76	0.63
LOA	-1.28, 0.35	-1.12, 0.40	-0.95, 0.31
n	196	193	161
*Mid infrarenal*			
Mean difference	-0.13 (-0.18, 0.07)	-0.08 (-0.1, -0.02)	-0.004 (-0.05, 0.04)
Variability	0.83	0.83	0.64
LOA	-0.95, 0.70	-0.91, 0.75	-0.65, 0.64
n	214	212	179
*Distal infrarenal*			
Mean difference	-0.07 (-0.1, -0.03)	-0.13 (-0.17, 0.09)	0.1 (0.06, 0.13)
Variability	0.49	0.61	0.49
LOA	-0.56, 0.42	-0.74, 0.48	-0.39, 0.59
n	215	209	179
*Max Coronal*			
Mean difference	-0.07 (-0.1, -0.03)	-0.05 (-0.09, -0.02)	0.05 (0.01, 0.08)
Variability	0.47	0.50	0.48
LOA	-0.54, 0.40	-0.56, 0.45	-0.44, 0.53
n	209	194	167

Measurement of the abdominal aorta were taken in the transverse plane at suprarenal, mid infrarenal and distal infrarenal sections and the maximal aortic measurement taken in the coronal plane. Mean difference (95% CI) (cm) of novice measurements from that of the assessors; variability of the mean difference; limits of agreement (LOA); n, number of interobserver pairs. Scans from which no measurement could be attained were discarded from the analysis and thus the numbers of inter-observer pairs are different in each aortic segment and each novice.

### Factors affecting performance

a. *Scanning Difficulties:* Both assessors and novices were able to capture and measure the aortic diameter in 92-100% of images taken. Novices had less confidence in their infrarenal measures; rating on average 18 scans (8.1%) as very difficult, compared with assessors’ whom rated only one infrarenal image (0.5%; *P* < .0001). Conversely, assessors rated more suprarenal scans as very difficult (8.4%) than the novice trainees (4.5%). There were no significant differences in measurement performance observed between scans that were rated as 1 to 3 on the difficulty scale.

b. *Body Habitus:* For both assessors and novices patient habitus was a significant factor contributing to scanning difficulty, as obese patients (BMI > 30 kg/m^2^) were more likely to have at least one image in their scan rated as a 4 on the difficulty scale, compared with their normal sized counterparts (41.8 vs. 17.6%; *P* = .0003). However, obesity and central adiposity did not affect the overall measurement accuracy of the novices.

c. *Aortic size:* The measures of variation between each of the novices and the assessors were generally greater for aortas ≥ 2.5 cm than those smaller. At the mid infrarenal level, the absolute mean difference of novice measurements was significantly greater in aortae measured 2.5 cm or greater (0.68 ± 0.48) compared with those taken in aortae with diameters less than 2.5 cm (0.22 ± 0.26; *P =* .001), this effect was also observed in distal infrarenal measurements (0.40 ± 0.50 vs. 0.19 ± 0.17; *P* = .0251) and in the maximal coronal measurements (0.29 ± 0.24 vs. 0.16 ± 0.15; *P* = .0067).

### Documenting progression in learning

**Figure 2:**
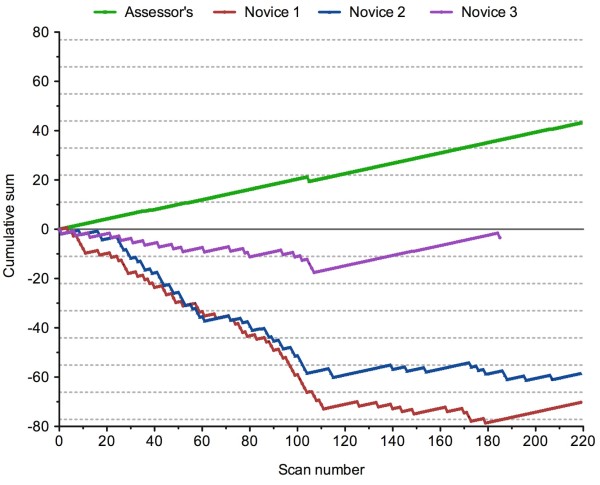
**Cusum plots demonstrating the progression in scanning efficiency for each novice over the study period.** The mean and SD were calculated from a sample of 220 scans with a 90% success rate, using 10,000 iterations in the bootstrapping procedure. Limit lines (dotted grey lines) demarcate when the success rate becomes higher or lower than would be expected if due to chance with 97.5% certainty. The criterion for a success was any scan completed within 10 min and is charted as an increase of 0.21 on the chart. Failure, results in a decrease on the plot of -1.79. Assessors have a higher success rate than 90% and cross the upper boundary limits at scans 56, 118, 171; Novice scanners have lower success rate than expected: Novice 1 crosses lower limits at scans 22, 39, 59, 83, 97,104, 173; Novice 2 crosses lower limits at scans 30, 44, 58, 90, 103; Novice 3 crosses lower limits at scan 81. After scan 110 a plateau in performance occurs in the novices performances.

a. *Cusum performance:* During the screening period the assessors completed 95% (205/216) of the scans within 5 minutes. Novice trainees achieved this in only 7.4% (48/653) of scans, and therefore this was not a useful learning benchmark. Using the 10-minute criterion with an expected 90% success rate as the benchmark, an improvement in the novice’s performance was seen over the course of the scanning period. Initially all novice trainees failed to achieve an acceptable success rate, but after scan 110 a plateau on the learning curve of all trainees was observed. Thereafter one trainee immediately and one at scan 180 showed performances that paralleled the assessors’ at this criteria, but the third had not yet reached this even by scan 210 (Figure 2).

b. *Learning progression:* After 110 scans the variability of novice measurements from the mid-infrarenal section of the aorta was lower than the first half of the screening period (-0.16 ± 0.34 vs. 0.01 ± 0.44; *P* < .0001). Little change in variability was observed with measurements in the more consistently measured coronal plane and distal transverse diameters. In the latter half of the screening period novices also had more confidence in their scanning ability, rating fewer images as 3 or 4 on the difficulty scale (29.4% vs. 37.5%; *P =* .03).

The two novices recruited subsequent to the initial study and assessed with the same quality control tools described yielded comparable outcomes on the Bland-Altman plots. Cusum was utilized in a similar manner to track learning progression.

## Discussion

The use of individuals with limited training as AAA screening sonographers remains a contentious issue. Whilst utilizing novices could significantly reduce the cost of screening programmes, the accuracy and reliability of abdominal aortic measurements is imperative. The novices in this study had a very high agreement with the skilled sonographers in the diagnosis of infrarenal AAA, and if a screening policy to refer very difficult scans for reevaluation in a vascular laboratory was applied, then all of the aneurysms would have been detected, excluding one aneurysm by a single novice.

Studies comparing CT and ultrasound measurements of the aorta commonly deem differences of less than 0.5 cm between the equivalent methods clinically acceptable [6,9,10,13,14]. This limit has also been adopted when evaluating the performance of newly trained sonographers. Five internal medicine doctors were able to measure the maximal aortic diameter within 0.5 cm of fully trained sonographers after 9.6 hours of training [7]. Kuhn and colleagues reported that Emergency Physicians could be trained to identify AAA after 3 days of ultrasound training. However, the measurement variability and reliability of the aortic measurements was not reported [15].

When imaging the maximal coronal aortic diameter, the novice’s measurements were within 0.5 cm 85-97% of the time, with a variability ranging between 0.47-0.50 cm. Measurements on the transverse plane were more variable than those taken from the coronal plane. These require more technically demanding steps such as the identification of the superior mesenteric and the renal arteries, a process which could generate greater variability in the site of measurement. This was particularly evident in the suprarenal and mid-infrarenal aortic measures. As illustrated by the Bland-Altman plots, novices had unacceptably high variability in these two sections, and showed a significant under sizing bias in the suprarenal aortic section. This suggests that for a screening programme employing novice trainees, rather than measuring the anterior-posterior diameter at discrete points along the aorta, a maximum measurement should be taken from a view that encompasses a continuous section of the aorta, such as a longitudinal or coronal measurement to the bifurcation point; a finding that is consistent with recommendations for AAA screeners in previous trials [16-19]. Additional training and feedback might have improved the quality of the transverse measurements as the ultrasound operators of the Singh et al. study [6] had substantially more training and practice and achieved acceptable accuracy of all the transverse aortic diameters.

Whatever measurement used, a continuing quality improvement programme should be required which monitors performance as well as the inclusion of safe guards in the screening protocol to reduce near misses. With this in mind, we looked at factors influencing performance. These included technical difficulties related to the subject’s habitus and clinical state in particular those who were obese. Similarly the study by Hoffman et al. [20] reported that visualization of the abdominal aorta largely depended on the sonographer’s experience, bowel gas and body mass index. However, despite the level of difficulty reported, the accuracy of aortic measurements by the novices were independent of obesity and central adiposity.

Other difficulties include the eccentric aneurysm and tortuous aortas, as well as the misidentification of other structures (e.g. the inferior vena cava, the superior mesenteric artery and the gall bladder) from the aorta and the accurate selection of the boundaries of the aortas [9,21]. It is suggested that these factors should all be specifically addressed in a training programme and in continuing quality improvement strategies.

Other safe guards to avoid missing aneurysms in these circumstances are the inclusion of protocols for referral of difficult subjects for a second assessment by an experienced sonographer. The decision to refer is dependent on the confidence of the screener to make the call whether an adequate study has or has not been performed. The novices in this study had a higher rate of referral for infrarenal scans than that of the assessors, and had less confidence in their measurements, however the novices also demonstrated a false confidence in their supra-renal measures, where they performed the worst. Monitoring the perceived difficulty scores for each scan as well as the referral rate for second assessments relative to an experienced sonographer may be good quality measures. Another proposal is to rescreen all 2.5-2.9 cm aortas by experienced sonographers [22]. This suggestion is compatible with Jaakola et al. [9] and our own result showing that measurements of aortas ≥ 2.5 cm were more variable than those smaller, however this approach compromises the cost-effectiveness of screening.

Another important factor in performance is the innate learning ability and individual systematic measurement bias of the individual screener. This study was able to identify some of these characteristics using the measures of variability, and Bland Altman plots as well as the perceptions of difficulty, visibility and the duration of the procedure. These measures could provide the basis for performance monitoring for individual feedback and remedial activities. They could be used for accreditation of training prior to independent screening as well as for continued practice audit to standardize the competence levels of novice-trained screeners.

The design of this study with one-on-one evaluation in over 200 scans might not be pragmatic for a training programme. Other approaches have been to evaluate quality of recorded images and accuracy of caliper placement [23]. However, these only address a small component of the screening task. Periodic direct supervision of screeners will play a part. These, however, are all time and resource intensive. Detection rates and abdominal aortic size profile of the screened cohorts are useful but require larger numbers to detect changes in performance of individual screeners. We have shown CUSUM analysis to be a useful and graphic tool for quality control. This sets a reasonable quality standard that the novice screener can be compared against to describe a scan-by-scan learning curve. CUSUM is a well established quality control tool used in industry and in clinical settings [24] and this approach has been used in assessing surgical training based on time to complete an appendicectomy [25]. In this study the improvement in scanning efficiency seen in the second half of the study was accompanied by more consistent measurement performance in the more difficult mid-infrarenal measurement and lower rates of self-reported difficult scans by the novices.

## Conclusion

Given 15 days of training including 50 ultrasound examinations, three novices were able to measure the maximal coronal diameter of the aorta for the purpose of AAA screening. The novices were less successful in measuring the segmental transverse diameters. For the purpose of using less experienced people as AAA screeners, it is recommended that effective quality improvement measures be implemented regularly to support the novices.

## Abbreviations

AAA: Abdominal aortic aneurysm; CAD: Clinically acceptable difference; LOA: Limits of agreement.

## Competing interests

All authors declare no competing interests.

## Authors’ contributions

ATVN collected the data, analysed and interpreted the data and contributed to the writing of the manuscript, including the critical revision of the manuscript. MPTV contributed to the analysis and interpretation of the data, as well as the writing and critical revision of the manuscript. GBH contributed to the conception and design of the project, the data collection and its analysis, and the critical revision of the article. IAT was involved in the conception and design of the project and the revision of the manuscript. AMvR was involved in the conception and design of the project, obtaining funding, the analysis and interpretation of the data, as well as the writing and critical revision of the manuscript. All authors read and approved the final manuscript.
